# Unique and facile solvothermal synthesis of mesoporous WO_3_ using a solid precursor and a surfactant template as a photoanode for visible-light-driven water oxidation

**DOI:** 10.1186/1556-276X-9-542

**Published:** 2014-10-02

**Authors:** Dong Li, Debraj Chandra, Kenji Saito, Tatsuto Yui, Masayuki Yagi

**Affiliations:** 1 Department of Materials Science and Technology, Faculty of Engineering, Niigata University, 8050 Ikarashi-2, Niigata 950-2181, Japan; 2 Precursory Research for Embryonic Science and Technology (PRESTO), Japan Science and Technology Agency (JST), 4-1-8 Honcho, Kawaguchi, Saitama 332-0012, Japan

**Keywords:** Tungsten trioxide, Mesoporous structure, Photoelectrocatalysis, Water oxidation

## Abstract

Mesoporous tungsten trioxide (WO_3_) was prepared from tungstic acid (H_2_WO_4_) as a tungsten precursor with dodecylamine (DDA) as a template to guide porosity of the nanostructure by a solvothermal technique. The WO_3_ sample (denoted as WO_3_-DDA) prepared with DDA was moulded on an electrode to yield efficient performance for visible-light-driven photoelectrochemical (PEC) water oxidation. Powder X-ray diffraction (XRD) data of the WO_3_-DDA sample calcined at 400°C indicate a crystalline framework of the mesoporous structure with disordered arrangement of pores. N_2_ physisorption studies show a Brunauer-Emmett-Teller (BET) surface area up to 57 m^2^ g^-1^ together with type IV isotherms and uniform distribution of a nanoscale pore size in the mesopore region. Scanning electron microscopy (SEM) images exhibit well-connected tiny spherical WO_3_ particles with a diameter of *ca.* 5 to 20 nm composing the mesoporous network. The WO_3_-DDA electrode generated photoanodic current density of 1.1 mA cm^-2^ at 1.0 V versus Ag/AgCl under visible light irradiation, which is about three times higher than that of the untemplated WO_3_. O_2_ (1.49 μmol; Faraday efficiency, 65.2%) was evolved during the 1-h photoelectrolysis for the WO_3_-DDA electrode under the conditions employed. The mesoporous electrode turned out to work more efficiently for visible-light-driven water oxidation relative to the untemplated WO_3_ electrode.

## Background

The recent advances in nanostructured materials have expanded their potential applications in much-desired materials for efficient solar energy conversion [1-6]. Photoelectrochemical (PEC) water splitting into oxygen and hydrogen is an attractive but challenging way for the conversion of solar energy, [7] following the pioneer work on a TiO_2_ photoanode for water splitting by Honda and Fujishima [8]. Unfortunately, owing to its wide electronic bandgap (3.0 to 3.2 eV), TiO_2_ absorbs only an ultraviolet fraction of a solar spectrum (which accounts for just 4% of solar irradiation), being consequently responsible for low efficiency in utilization of solar light [2,7,9]. For solar water splitting, intensive researches have been focused on nanostructured materials with narrow bandgaps including WO_3_[3,4,10-19]. WO_3_, an n-type semiconductor, has attracted immense attention as a photoanode material for water oxidation in PEC cells because of its visible light response (bandgap, *E*_g_ = 2.6 to 2.8 eV), a valence band edge position thermodynamically possible for water oxidation (about 3 V versus the normal hydrogen electrode), and good photochemical stability under the acidic conditions [3,10-12,20-24].

Porous material design, which has been developed employing template-directed approaches using small organic compounds [25], supramolecular assembly [26], and polymer beads [27], is of great importance in many research fields because of the high porosity, large area per unit volume, and favorable design of a porous structure [25,28,29]. So far, several efforts in nanostructural and porosity controls of WO_3_ have been provided to increase the contact area between an electrode and an electrolyte solution and to make electron transport in WO_3_ films more efficient, enhancing performance of PEC water oxidation at WO_3_ electrodes [3,11,30-33]. For example, Santato et al. have reported that crystalline WO_3_ photoanodes with interconnected nanoparticulate structures improved photoelectrochemical properties [30-32]. Berger et al. have demonstrated that random porous layers of WO_3_ produced significantly higher photocurrent efficiency than a compact layer [33]. Our group recently demonstrated a crystalline small mesoporous network of a WO_3_ photoanode for high improvement in performance of PEC water oxidation [3].

Numerous methods have been employed to control the dimension, morphology, and crystal structure of WO_3_, e.g., vacuum evaporation [34], chemical vapor deposition [35,36], sol–gel precipitation [22,30-32], hydrothermal/solvothermal [37-40], surfactant/hard template techniques [3,41,42], and so on. Among the abundant methods, hydrothermal/solvothermal techniques can provide a cost-effective and one-step route synthesis of WO_3_[37-40]. Although the surfactant template techniques require a liquid tungsten precursor to utilize interaction with a surfactant in principle, we have focused on the interaction between a solid tungsten precursor and a surfactant under solvothermal conditions to yield mesoporous WO_3_. Herein, we report the unique and facile synthesis of mesoporous WO_3_ utilizing solid H_2_WO_4_ as a tungsten precursor with an organic amphiphilic molecule, dodecylamine (DDA), as a surfactant template for porosity of the nanostructure. The mesoporous WO_3_ exhibited high surface area and improved the performance of PEC water oxidation compared to the corresponding materials prepared without a template.

## Methods

### Materials

Tungstic acid (H_2_WO_4_) was purchased from Kanto Chemical Co., Inc. (Chuo-ku, Tokyo, Japan). DDA was obtained from Sigma-Aldrich (St. Louis, MO, USA). Polyethylene glycol (PEG, molecular weight = 2,000) was obtained from Wako Chemical Co. (Osaka, Japan). Marpolose (60MP-50) was purchased from Matsumoto Yushi-Seiyaku Co. (Osaka, Japan). An indium tin oxide (ITO)-coated glass substrate was obtained from Asahi Glass Co. (Tokyo, Japan). Millipore water (Merck Ltd., Tokyo, Japan) was used for all the experiments. All other chemicals unless mentioned otherwise were of analytical grade and used as received.

### Synthesis of mesoporous WO_3_

In a typical synthesis, 1.7 g of DDA (9.0 mmol) was dissolved in 15 mL ethanol under stirring at room temperature. Tungstic acid (0.9 g; 3.6 mmol) was added to the DDA solution with stirring for 30 min to yield a suspension. It was transferred to a Teflon-lined stainless steel autoclave and then placed in an oil bath at 150°C for 24 h. After the autoclave was cooled down to room temperature, the solid product was recovered by centrifugation, then washed repeatedly by ethanol and air-dried. The solid product was calcined at 400°C with a rate of 1°C min^-1^ and then maintained at 400°C for 1 h in flowing N_2_, followed by changing to O_2_ flow (at 400°C) for 2 h to result in a WO_3_ sample (denoted as WO_3_-DDA). A control sample (denoted as WO_3_-bulk) was prepared in the same manner except for the addition of DDA.

### Preparation of electrodes

The WO_3_ film-coated ITO electrodes (ITO/WO_3_) were prepared employing a doctor-blade technique. Before coating, ITO glass substrates (1.0 cm^-2^ area) were cleaned up by a UV-ozone treatment (photo surface processor PL16-110, Sen Lights Co., Osaka, Japan) for 15 min. In a typical procedure, WO_3_ powder (200 mg), PEG (100 mg), and Marpolose (20 mg) were mixed in 300 μL of water. The mixture suspension was stirred for approximately 2 to 4 h until a smooth paste was formed. The resulting paste was squeezed over an ITO glass substrate by a doctor-blade coater and dried at 80°C for 15 min. After repeating the procedure for two times, the electrodes were calcined at 400°C and maintained at 400°C in flowing N_2_ for 1 h, followed by changing to O_2_ flow (at 400°C) for 2 h.

### Structural characterization

Characterization of the morphological features and the crystalline phase was conducted by field-emission scanning electron microscopy (FESEM; JSM-6500 F, JEOL Ltd., Akishima, Tokyo, Japan) and powder X-ray diffraction (XRD; MiniFlexII, Rigaku Corporation, Tokyo, Japan) using monochromated Cu Kα (*λ* = 1.54 Å) radiation. Nitrogen adsorption-desorption isotherms were measured using a BELSORP-miniII (BEL Japan, Inc., Osaka, Japan) at 77 K. Prior to gas adsorption, samples were degassed in vacuum for 4 h at 150°C. The Brunauer-Emmett-Teller (BET) method was utilized to calculate the surface areas. The pore size distributions were obtained from analysis of the adsorption branches of the isotherms by the Barrett-Joyner-Halenda (BJH) method. Fourier transform infrared spectra were recorded on a Jasco FT/IR-4200 spectrophotometer (Jasco Inc., Tokyo, Japan).

### Photoelectrochemical measurements

Photoelectrochemical measurement was carried out in a two-compartment photoelectrochemical cell separated by a Nafion membrane using an electrochemical analyzer (HZ-3000, Hokuto Denko Co. Ltd., Tokyo, Japan). A three-electrode system has been employed by using ITO/WO_3_ and Ag/AgCl electrodes in one compartment as the working and reference electrodes, respectively, and a Pt wire in the other compartment as the counter electrode. An aqueous 0.1 M phosphate solution was used as an electrolyte in both compartments of the cell, which was saturated with Ar gas prior to the measurement. The cyclic voltammogram (CV) was recorded at a scan rate of 50 mV s^-1^ at 25°C. Light (*λ* > 390 nm) was irradiated from the backside of the working electrode using a 500-W xenon lamp (Optical ModuleX; Ushio Inc., Tokyo, Japan) with a UV-cut filter (L39) and liquid filter (0.2 M CuSO_4_) for cutting of heat ray. The output of light intensity was calibrated as 100 mW cm^-2^ using a spectroradiometer (USR-40; Ushio Inc., Tokyo, Japan). Photoelectrocatalysis was conducted under the potentiostatic conditions of 0.5 V versus Ag/AgCl at 25°C under illumination of light (*λ* > 390 nm, 100 mW cm^-2^) for 1 h. The amounts of H_2_ and O_2_ evolved were determined from the analysis of the gas phase (headspace volume: 87.3 mL) of counter and working electrode compartments, respectively, using gas chromatography (GC-8A with a TCD detector and molecular sieve 5A column and Ar carrier gas; Shimadzu Corporation, Kyoto, Japan).

## Results and discussion

**Figure 1:**
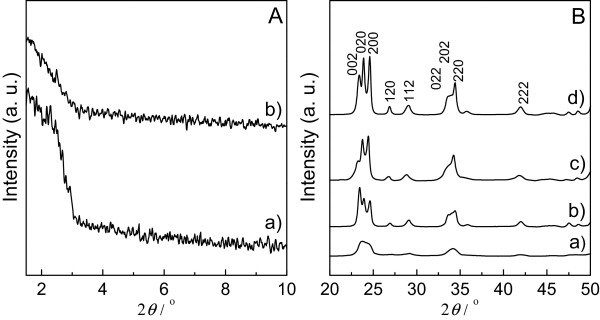
**Small-angle and wide-angle X-ray diffraction patterns. (A)** Small-angle and **(B)** wide-angle XRD patterns of WO_3_-DDA and WO_3_-bulk samples after being calcined at 400°C and 500°C. **(a)** WO_3_-DDA calcined at 400°C, **(b)** WO_3_-DDA calcined at 500°C, **(c)** WO_3_-bulk calcined at 400°C, and **(d)** WO_3_-bulk calcined at 500°C.

The powder XRD patterns of the WO_3_ samples calcined at 400°C and 500°C are shown in Figure 1. Small-angle XRD patterns (Figure 1 (a)) of WO_3_-DDA at 400°C showed a single diffraction peak at low 2*θ*, being a sign of formation of mesoporous structures, but the weak intensity and broadness of the peak are possibly due to disordered mesoporous structures. The *d*-spacing, calculated from the XRD peak at 2*θ* = 2.3° is 3.78 nm. Weakening of the intensity of the diffraction peak for WO_3_-DDA at 500°C (Figure 1 (b)) suggests degradation of the mesostructure at higher temperature. The wide-angle XRD patterns of both the WO_3_-DDA and WO_3_-bulk samples revealed crystallization of the framework after calcination at 400°C and higher degree of crystallization at 500°C, though crystallinity of WO_3_-bulk seems to be higher than that of WO_3_-DDA at both calcination temperatures. The *d*-spacings calculated from the XRD peaks of both WO_3_-DDA and WO_3_-bulk were in good agreement with phase-pure monoclinic WO_3_ (JCPDS number: 43–1305). Average crystallite sizes for WO_3_-DDA, estimated using [002] reflections were 5.7 and 11.6 nm at 400°C and 500°C, respectively, which suggests that progressive growth of the WO_3_ nanocrystal in the porous network is responsible for degradation of the mesostructure at 500°C.

**Figure 2:**
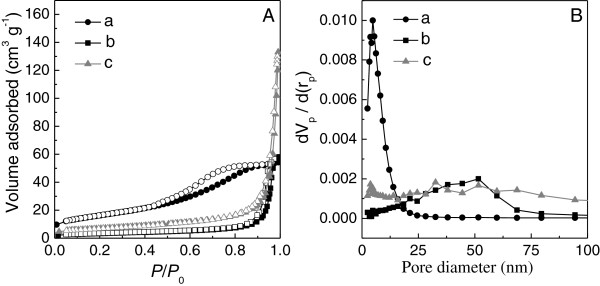
**N**_**2 **_**sorption isotherms and pore size distribution. (A)** N_2_ sorption isotherms and **(B)** pore size distribution of WO_3_-DDA and WO_3_-bulk samples after being calcined at 400°C and 500°C. In N_2_ sorption, isotherm adsorption and desorption points are marked by filled and empty symbols, respectively. **(a)** WO_3_-DDA calcined at 400°C, **(b)** WO_3_-DDA calcined at 500°C, and **(c)** WO_3_-bulk calcined at 400°C.

N_2_ adsorption/desorption isotherms of the WO_3_ samples calcined at 400°C and 500°C are shown in Figure 2. The isotherm (Figure 2 (a)) of WO_3_-DDA calcined at 400°C could be classified as type IV, characteristic of mesoporous materials [26,43]. In this isotherm, the adsorption amount gradually increased in a range of *P*/*P*_0_ = 0.4 to 0.85, which could be explained by the classical capillary condensation observed for mesopores. The H2 hysteresis loop in the isotherm (Figure 2 (a)) may be caused by roughness of the pore and particle surface [44]. The BET surface area and mesopore volume for WO_3_-DDA calcined at 400°C were 57 m^2^ g^-1^ and 0.08 cm^3^ g^-1^, respectively, as summarized in Table 1. The pore size distribution (Figure 2) by the BJH method shows narrow distribution with a peak pore width at 4.9 nm. Isotherm of WO_3_-DDA calcined at 500°C shows a predominantly type II nature, and the BET surface area was drastically reduced to 12 m^2^ g^-1^. The pore size distribution of WO_3_-DDA calcined at 500°C gives a wider peak at approximately 50 nm due to large interparticle pores. These results are in accordance with the degradation of the mesoporous structure of WO_3_-DDA due to progressive growth of WO_3_ nanocrystals at higher temperature of 500°C, as observed in the XRD measurement. The WO_3_-bulk sample synthesized without DDA exhibited typical type II isotherms, characteristic of nonporous solids. The BET surface area is 24 m^2^ g^-1^ at 400°C, which is noticeably low compared to the mesoporous WO_3_-DDA.

**Table 1:** **Physicochemical properties of WO**_**3 **_**samples**

**Sample name**	**Calcination temperature (°C)**	***d*-spacing (nm)**	**Surface area (m**^**2**^ **g**^**-1**^**)**	**Pore volume (cm**^**3**^ **g**^**-1**^**)**	**Pore size (nm)**
WO_3_-DDA	400	1.70	57	0.08	4.9
WO_3_-DDA	500	-	12	0.09	48.2
WO_3_-bulk	400	-	24	0.19	52.7

**Figure 3:**
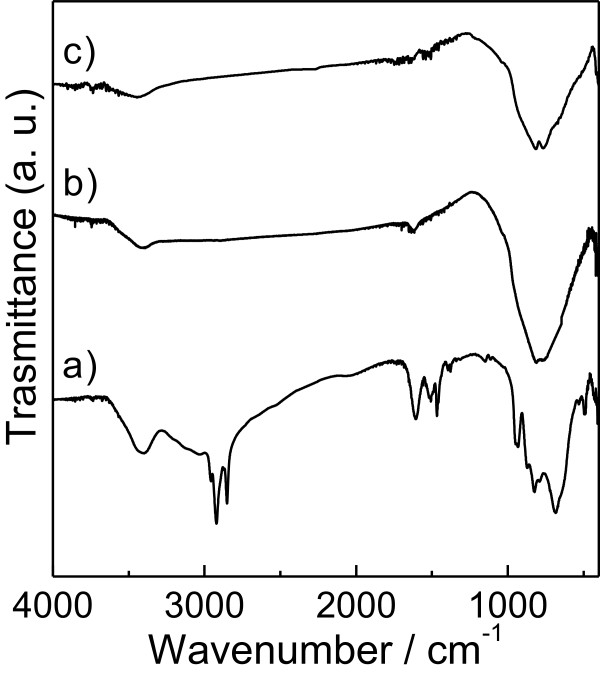
**FTIR spectra of WO**_**3**_**-DDA samples. (a)** The as-made sample and samples after being calcined at **(b)** 400°C and **(c)** 500°C.

The Fourier transform infrared (FTIR) spectra of as-made (before calcination) and calcined (400°C and 500°C) WO_3_-DDA samples are shown in Figure 3. C-H stretching vibration bands of the hydrocarbon chains at 2,919 cm^-1^ (asymmetric) and 2,844 cm^-1^ (symmetric) along with C-H bending vibration bands at 1,469 cm^-1^ of CH_2_ groups were clearly observed in the as-made sample. Comparing the FTIR spectra of the as-made WO_3_-DDA with calcined WO_3_-DDA samples, we could see that peaks due to C-H vibration diminished completely for the calcined samples. This indicates complete removal of DDA during calcination at 400°C and 500°C, which is very much necessary to generate high porosity for these mesoporous materials.

**Figure 4:**
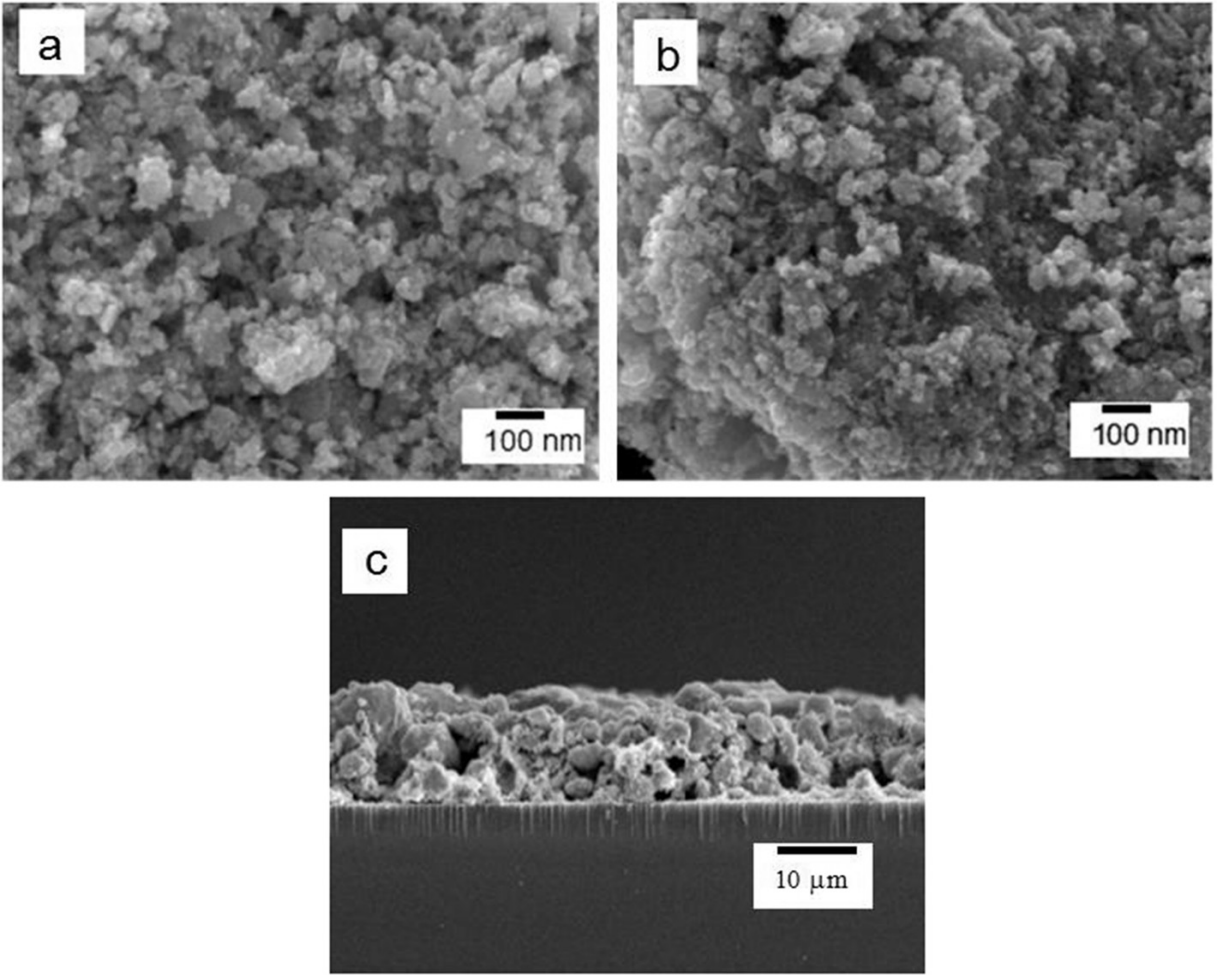
**Scanning electron microscopic (SEM) images.** Top view of WO_3_-DDA samples calcined at **(a)** 400°C and **(b)** 500°C. **(c)** Cross-sectional view of the ITO/WO_3_-DDA electrode after being calcined at 500°C.

The scanning electron microscopy (SEM) images of the calcined WO_3_-DDA samples are shown in Figure 4. The SEM images of the top view (Figure 4a,b) exhibit that a mesoporous network is composed of tiny spherical WO_3_ particles of *ca.* 5 to 20 nm in diameter, being well connected to each other. In a few places, the spherical particles agglomerate to form large particles. A close look into these images suggests that the average dimension of particles increases with calcination temperature from 400°C to 500°C due to sintering of WO_3_ nanocrystals at higher calcination temperature. After preparation of a mesoporous WO_3_ film on an ITO electrode, the film thickness was measured to be *ca.* 12 μm from the cross-sectional SEM image (Figure 4c). This crystalline mesoporous structure of the connected WO_3_ particles is important to yield a large interface between the electrolyte and film as well as efficient electron transport through the film, which are consequently expected to work efficiently for PEC water oxidation since the electron and hole pairs generated by photoexcitation of WO_3_ would have less chance to recombine before participating in a water oxidation reaction at the WO_3_ surface.

**Figure 5:**
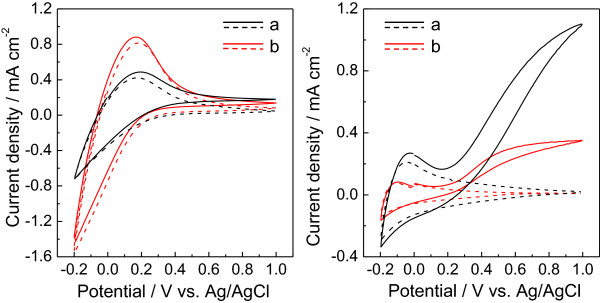
**Cyclic voltammograms (CVs) for samples calcined at 400°C (left) and 500°C (right). (a)** ITO/WO_3_-DDA and **(b)** ITO/WO_3_-bulk electrodes in a 0.1 M phosphate buffer solution with pH = 6.0. The dashed and solid lines represent CVs measured in the dark and upon irradiation of visible light (*λ* > 390 nm, 100 mW cm^-2^), respectively.

The PEC properties of the ITO/WO_3_ electrodes were studied in a 0.1 M phosphate solution. Figure 5 shows the CVs of the ITO/WO_3_ electrodes. On CVs of samples calcined at 400°C for both WO_3_-DDA and WO_3_-bulk (Figure 5, left), no redox response was observed in the dark in a potential range of 0.4 ~ 1.0 V versus Ag/AgCl except for a response based on WO_3_/H_x_WO_3_ below 0.2 V. Upon irradiation of visible light, the anodic current (0.13 ~ 0.18 mA cm^-2^ at 1.0 V versus Ag/AgCl) was hardly generated for both samples. This is ascribed to insufficient crystallinity of both WO_3_-DDA and WO_3_-bulk calcined at 400°C. Crystallinity rather than porosity for samples calcined at 400°C is a dominant factor for the PEC performance of the WO_3_-based photoanode under the conditions employed [20,32]. On CVs of the samples calcined at 500°C (Figure 5, right), the significantly high photoanodic current due to water oxidation was observed upon visible light irradiation above an onset potential of 0.17 V versus Ag/AgCl due to higher crystallinity. The photoanodic current reached 1.1 mA cm^-2^ at 1.0 V for WO_3_-DDA, which is about three times higher compared to that for the WO_3_-bulk (0.36 mA cm^-2^ at 1.0 V) electrode in spite of the degradation of mesoporous structure for WO_3_-DDA calcined at 500°C. The degraded mesoporous structure for WO_3_-DDA might result in favorable conditions for PEC water oxidation compared with the nanoparticle structure of WO_3_-bulk. Otherwise, another important factor might be involved in the higher performance of the WO_3_-DDA electrode. In the present paper, we do not pursue interpretation of the higher performance of the WO_3_-DDA electrode because our attention is on the solvothermal synthesis of a mesoporous structure of WO_3_.

**Figure 6:**
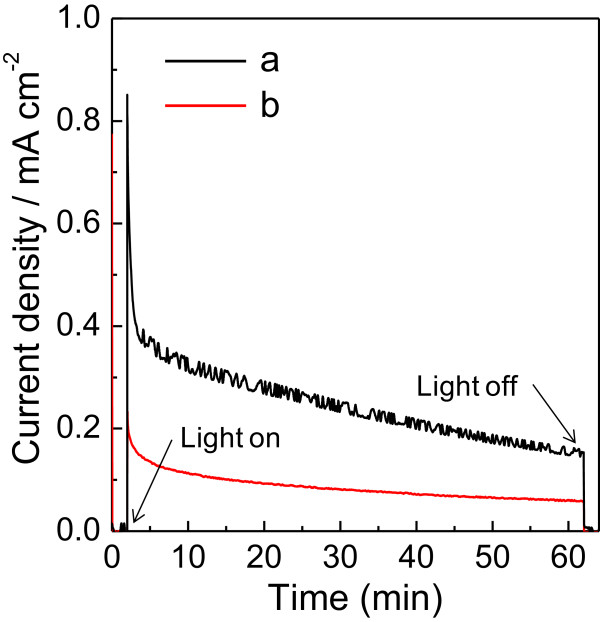
**Photocurrent density versus time profiles during PEC water oxidation using samples calcined at 500°C. (a)** ITO/WO_3_-DDA and **(b)** ITO/WO_3_-bulk electrodes in 0.1 M phosphate buffer solution of pH = 6.0 at 0.5 V versus Ag/AgCl with visible light irradiation (*λ* > 390 nm, 100 mW cm^-2^).

Photoelectrocatalysis over the ITO/WO_3_ electrodes was conducted in a 0.1 M phosphate solution (pH = 6.0) under potentiostatic conditions at 0.5 V versus Ag/AgCl for 1 h upon visible light irradiation (*λ* > 390 nm, 100 mW cm^-2^). The photocurrent-time profiles of both WO_3_-DDA and WO_3_-bulk calcined at 500°C exhibit initial spikes in the photocurrent upon illumination (related with the capacitance component at the solid–liquid interface), followed by a photocatalytic current, as shown in Figure 6. The photocurrent density of WO_3_-DDA at 1 min was 0.37 mA cm^-2^, which is 2.5 times higher than that of the WO_3_-bulk (0.15 mA cm^-2^ at 1 min) electrode. The charge amount passed during 1-h photoelectrocatalysis for WO_3_-DDA (0.89 C) was 3.9 times higher than that of WO_3_-bulk (0.23 C). As a consequence of the high charge amount, the markedly high amount (*n*_O2_ = 1.5 μmol, Faradaic efficiency (FE_O2_) = 65%) of O_2_ evolved for the WO_3_-DDA electrode compared to that (*n*_O2_ = 0.4 μmol, FE_O2_ = 58%) of the WO_3_-bulk electrode, as summarized in Table 2. As compared with performances of PEC water oxidation under the same conditions for WO_3_-based photoanodes reported earlier [3], the performance of the present mesoporous WO_3_-DDA is lower than that of the small mesoporous WO_3_ film (*n*_O2_ = 4.2 μmol, FE_O2_ = 79%) [3], but much higher than that of interparticle mesoporous WO_3_ (*n*_O2_ = 0.9 μmol, FE_O2_ = 61%) and bulk WO_3_ (*n*_O2_ = 0.4 μmol, FE_O2_ = 44%) [3]. The high performance of the mesoporous WO_3_-DDA photoanode is attributed to its high surface-to-volume ratio which offers a large number of water oxidation sites at the electrolyte-WO_3_ interface, and well-connected WO_3_ particles for efficient electron transport through the film.

**Table 2:** **Summary of photoelectrocatalytic water oxidation at different ITO/WO**_**3 **_**photoanodes calcined at 500°C in 0.1 M phosphate solution**

**Sample name**	**Charge (C)**	***n***_**O2 **_**(μmol)**	**FE**_**O2**_^**a **^**(%)**	***n***_**H2**_^**b **^**(μmol)**	**FE**_**H2**_^**c **^**(%)**
WO_3_-DDA	0.89	1.49	65.2	4.46	97.4
WO_3_-bulk	0.23	0.35	58.1	0.89	74.3

^a^Faradic efficiency of O_2_ evolution. ^b^*n*_H2_ is the amount of H_2_ evolved in the Pt counter electrode compartment. ^c^Faradic efficiency of H_2_ evolution.

## Conclusions

We have prepared mesoporous WO_3_ materials by a unique and facile solvothermal method using solid H_2_WO_4_ as a tungsten precursor. DDA was used as a template for the formation of nanostructure, which generates mesoporosity after removing DDA by calcination. The present surfactant template technique is very unique in terms of use of a solid tungsten precursor in a solvothermal method, compared with a common technique using liquid tungsten precursors for interaction with surfactants in principle. The mesoporous network has a disordered arrangement of pores which is composed of well-connected tiny spherical WO_3_ particles with a diameter of *ca.* 5 to 20 nm. The DDA-templated WO_3_ photoanode showed three times higher photoanodic current density upon visible light irradiation and provided the efficient performance of PEC water oxidation compared to the untemplated WO_3_, which is promising as an efficient material for high-performance solar energy conversion.

## Competing interests

The authors declare that they have no competing interests.

## Authors’ contributions

LD prepared the samples and performed the photoelectrochemical measurements. DC carried out the analysis and optimization of the results and drafted the manuscript. KS and TY helped analyze the results. MY supervised the data analysis and interpretation of the results and helped draft the manuscript. All authors read and approved the final manuscript.
